# In search of Schrödinger’s patch: a functional approach to habitat delineation

**DOI:** 10.1007/s10980-025-02291-x

**Published:** 2026-01-21

**Authors:** Matthew Dennis, Jonathan Huck, Claire Holt, Ewan McHenry, Erik Andersson, Sonali Sharma, Dagmar Haase

**Affiliations:** 1 CIRCLE, Department of Geography, University of Manchester, Manchester, UK; 2 Department of Science, Natural Resources & Outdoor Studies, University of Cumbria, Rydal Rd, Ambleside, UK; 3 Woodland Trust, Grantham, Lincolnshire UK; 4 Faculty of Biological and Environmental Sciences, Department of Environmental Sciences & Helsinki Institute of Sustainability Science, University of Helsinki, Helsinki, Finland; 5 Department of Geography, Lab for Urban Ecology, Humboldt University Berlin, Rudower Chaussee 16, 12489 Berlin, Germany; 6 Department of Computational Landscape Ecology, Helmholtz Centre for Environmental Research–UFZ, Leipzig, Germany; 7 Stockholm Resilience Centre, Stockholm University, Stockholm, Sweden

**Keywords:** Habitat patch, Fragmentation, Biodiversity, Landscape modelling, Fuzzy set theory, Spatial ecology

## Abstract

**Context:**

The effective delineation of habitat is crucial for understanding drivers of habitat loss and fragmentation, and their effects on biodiversity outcomes at local to global scales. The concept of the habitat patch is central to this process but presents both theoretical and methodological challenges related to the seemingly irreconcilable tendency of habitat to simultaneously exhibit characteristics of both gradation and aggregation. This apparent contradiction, recently described as the *continuity-contiguity problem* in landscape ecology, presents a problem of classification in which the associated ambivalence is analogous to that surrounding the fate of Schrödinger’s Cat.

**Objectives:**

This is the first of a pair of papers that aim to address the theoretical and methodological challenges associated with the habitat patch concept. This first paper aims to (a) articulate the theoretical and practical limitations of working with the habitat patch concept and (b) set out a framework based on a functional definition of habitat that captures the tendency of resources to exhibit both discrete and continuous spatial characteristics. The second paper (Dennis et al. this issue) presents a demonstration of this framework applied to a real-world landscape, in which the impact of adopting alternative perspectives on habitat delineation on potential functional connectivity is revealed.

**Methods:**

We present a new methodological approach that integrates alternative gradient and patch-based models of habitat in landscape ecology. We achieve this integration by leveraging the notion of geographical vagueness and the application of fuzzy set theory to land cover classification. We apply this approach to simulated landscapes that contain information on membership values to different land cover classes and their associated uncertainty. We then demonstrate the functional delineation of habitat from these landscapes based on the use of species-specific parameters, the leveraging of spatial kernels, and *type-1* and *type-2* fuzzy sets. The possibility of incorporating this approach into subsequent workflows is then described using estimates of between-patch distances and potential functional connectivity as examples.

**Results:**

Our method provides a functional spatial delineation of habitat that reflects both resource-based and patch-based habitat perspectives and can be applied to any gradient or patch-based landscape modelling method. This approach achieves the integration of multiple resource types, habitat complementarity associated with neighbouring cover types, and negative edge effects. We refer to this measure of habitat as *Functional Habitat* so-called as it reflects the total availability of habitat accounting for the influence of all land cover types and positive and negative neighbourhood effects.

**Conclusion:**

This paper describes a functional approach to habitat delineation and its integration into the computation of fragmentation-related metrics. This methodological framework achieves, for the first time, (1) a multivariate delineation of habitat based on *type-1* fuzzy membership and the operationalising of neighbourhood effects and (2) the harnessing of uncertainty in land cover classification (*type-2* fuzzy membership) to achieve a distribution of possible outcomes that resolves the *continuity-contiguity problem*. This new methodology provides a long-awaited functional definition of habitat patches for those seeking to understand the role of habitat fragmentation in biodiversity outcomes.

## Introduction

The development of effective approaches to the spatial delineation of habitat is both necessary for answering a range of questions in biodiversity studies and challenging from a methodological perspective. Necessary, because key drivers of change in ecological communities, such as habitat loss and fragmentation, are spatially-structured processes; challenging, due to the seeming irreconcilability of the tendency of habitat resources to exhibit characteristics of both gradation and aggregation. This tension has been the topic of previous debate (Dennis et al. 2007, 2014; Vanreusel and Van Dyck 2007) but methods to navigate this tension have not been forthcoming. At the centre of this apparent contradiction is the concept of the habitat patch and the question of its suitability for investigating ecological processes that influence biodiversity outcomes (Fahrig 2013; Dennis and Huck 2025). In this paper, which is the first of two addressing this problem (along with Dennis et al., this issue), we first articulate the theoretical and practical limitations of working with the habitat patch concept. We then set out a framework based on a functional definition of habitat that captures the tendency of resources to exhibit both discrete and continuous spatial characteristics. The second paper (Dennis et al., this issue) provides a demonstration of this framework applied to a real-world case study, highlighting the potential use of a functional delineation of habitat in fragmentation-biodiversity studies.

The concept of the habitat patch is central to research on the influence of fragmentation on biodiversity outcomes but has continued to receive criticism with respect to its ability to effectively and realistically characterize the distribution (and definition) of habitat within a so-called matrix of non-habitat (Fahrig 2013; Dennis et al. 2014, 2024a; Dexheimer and Despland 2023). Dennis and Huck (2025) recently presented the various criticisms of the patch concept as instances of the *continuity-contiguity problem*. This problem derives from the prevalence in landscape research of adopting either a binary representation of habitat (versus non-habitat with no intermediate classes) or, alternatively, a continuous representation of habitat (such that habitat does not occur in patches but where different cover types intergrade without discrete boundaries). This bifurcation of habitat into either contiguous or continuous space presents a major obstacle to understanding the true ecological effects or habitat fragmentation. This is because neither of these perspectives is by itself equipped to capture real-world processes. The contiguous (binary) perspective offers a geometric understanding of habitat and is predicated on an area-based formulation where the size of habitat polygons is assumed to be equal to resource availability. However, this binary view does not reflect the heterogenous nature of resource distributions that culminate in habitat provision. On the other hand, a continuous perspective that allows habitat to grade into adjacent cover types is more consistent with the observation that matrix quality influences overall resource provision (Gallé et al. 2022). Notwithstanding its ability to capture such transitions, this perspective ignores the tendency of habitat to aggregate in space, a characteristic that underpins theoretical positions related to the role of hierarchies (Wu 2013) and discontinuities (Holling 1992), as well as methodological approaches such as landscape metrics (McGarigal and Marks 1995) and connectivity indices (Keeley et al. 2021). An appreciation of both continuous and contiguous properties of habitat is particularly important for effective hypothesis testing within fragmentation-biodiversity research. Whether or not habitat is defined better as a distribution or an aggregation of resources is at the centre of the debate around the influence of fragmentation on biodiversity outcomes. This is because the degree to which habitat exhibits aggregation is inseparable from the notion of fragmentation itself.

Others have gone so far as to claim that the concept of the patch may be unnecessary and that only the amount of habitat within an appropriate distance of a given location is needed to explain local biodiversity outcomes (Fahrig 2013). For example, the habitat amount hypothesis supposes that fragmentation, and relatedly, configuration, has a negligible influence on biodiversity outcomes where only the amount of habitat within a given landscape is relevant (Fahrig 2013). However, even hypotheses that negate the relevance of the habitat patch do not avoid the need to delineate habitat in space. This is because the testing of such hypotheses still necessitates the delineation of contiguous patches of habitat (in order to reject the null hypothesis that fragmentation *is* relevant). In such studies, a binary perspective is usually adopted, whereby each location is classified either as ‘habitat’ or ‘not habitat’, typically based on a single land cover type (e.g. Evju and Sverdrup-Thygeson 2016; Lindgrem and Cousins 2017; Melo et al. 2017; Watling et al. 2020). Hence, even in the attempt to dismiss the patch concept, issues around the spatial delineation of habitat persist.

Two other issues stem from the conceptual and methodological ambiguity surrounding the habitat patch: the *gap crossing problem* and the *multivariate habitat problem* (Dennis and Huck 2025). The gap-crossing problem refers to the question of whether two seemingly discrete patches in close proximity to each other should be delimited as one or two patches. This problem occurs when a binary partitioning of the landscape between habitat patches and the surrounding matrix is adopted without consideration of the quality of both the delineated habitat patch and the intervening resources or the mobility of the species under investigation. It arises primarily from the unlikely assumptions that (a) a discrete habitat-matrix classification is sufficient to anticipate species movement and resource use and (b) locations classified as habitat are of sufficient quality that movement cost within the patch is negligible (Watts and Handley 2010). Hence, we argue that the gap-crossing problem has a classification-based, rather than an ecological, derivation. The multivariate habitat problem arises from the convention of associating single land cover types with habitat and the challenge of incorporating multiple cover types into the delineation of a spatially discrete phenomenon. The assumption that habitat amount is equal to the area covered by a single biotope (i.e. a single vegetation cover type) also removes the possibility that spatial context may influence habitat amount or quality. For example, habitat complementarity, where species exploit multiple resources in a landscape, occurs as a function of the presence of primary habitat and the ability to access nearby complementary resources. Such a definition, though theoretically supported (Hartemink et al. 2015; Turlure et al. 2019), is poorly reflected in conventional methods of habitat delineation. These assumptions prevent the development of more functional approaches to habitat delineation. The move from structural to more functional methods of landscape analysis has been a key theme in fragmentation studies where connectivity is concerned (Saura and Pascual- Hortal 2007; Watts and Handley 2010; Dennis et al. 2024a). An approach to habitat delineation whereby habitat provision emerges from the properties of contributing resources (e.g. their spatial extent, surrounding context and quality) and species requirements, is therefore well-aligned with a process-oriented approach (Fletcher and Fortin 2018). The development of such a method must consider the continuous nature of resource distributions as well as their tendency to aggregate in space. This is the central methodological challenge associated with the *continuity-contiguity problem* that prevents a gradient view of habitat from being reconciled with the need to model topographic relationships (e.g. effective distances and connectivity).

### The need for a functional spatial ecology

Several authors have attempted to view patch delineation from more functional standpoints. For example, Halstead et al. (2019) used stacked species distribution models (SDMs) to estimate community-level habitat availability. They report improved model performance when predicting species richness as a result of using a more species-specific (i.e., functional) measure of habitat. However, correlative modelling of species-specific habitat suitability is still subject to the *continuity-contiguity problem*. This is because, regardless of whether they are based on classified land cover datasets or continuous data, SDMs typically require arbitrary thresholds to delineate habitat patches for use in further analysis (of fragmentation effects, for example). Similarly, although the influence of nearby cover types can be integrated into SDMs through focal analyses or distance-based measures, these typically rely on a Boolean scheme to delineate relevant features in the landscape (such as roads or water bodies). Hence, habitat models derived from SDMs do not present a complete framework for patch delineation that avoids the *continuity-contiguity problem*.

Outside of landscape ecology, much interest in the development of more sophisticated approaches to patch delineation has come from the field of geographical information science (GIS). For example, significant advances have been made in fuzzy land cover representation, where boundaries between landscape elements are gradual as opposed to fixed in traditional binary classification schemes (Arnot et al. 2004; Fisher et al. 2006; Comber et al. 2010). Such work has provided a means of differentiating landscape components, supported by geographical theory.

Consider the fuzzy classification approach in which a single membership value within the continuous scale $[\mathrm{0,1}]$ represents the degree to which a given pixel is a member of each of a set of possible land cover classes, which is known as a *type-1* fuzzy set. These values are typically used to determine the extent of a given class in the landscape using a thresholding approach known as an alpha cut ($\alpha$-cut), whereby all pixels with a membership value $\ge \alpha$ are included in the class and the remainder are excluded (Arnot and Fisher 2007). This process, however, results in a Boolean set, which still fails to capture the geographical vagueness inherent in the patch and prevents the integration of other resource types into habitat delineation. Therefore, the end point of a *type-1* fuzzy classification still does not reflect a mechanistic understanding of how biotopes transition across space, nor is its derivation empirically driven. The challenge therefore remains to bridge this mechanistic understanding of habitat transitions and the need to identify topographic relationships in space. Overcoming the current methodological impasse towards reconciling these two views would imply a significant step forward in the development of a functional spatial ecology.

### Incorporating fuzzy and landscape ecology methods and theory

**Fig. 1:**
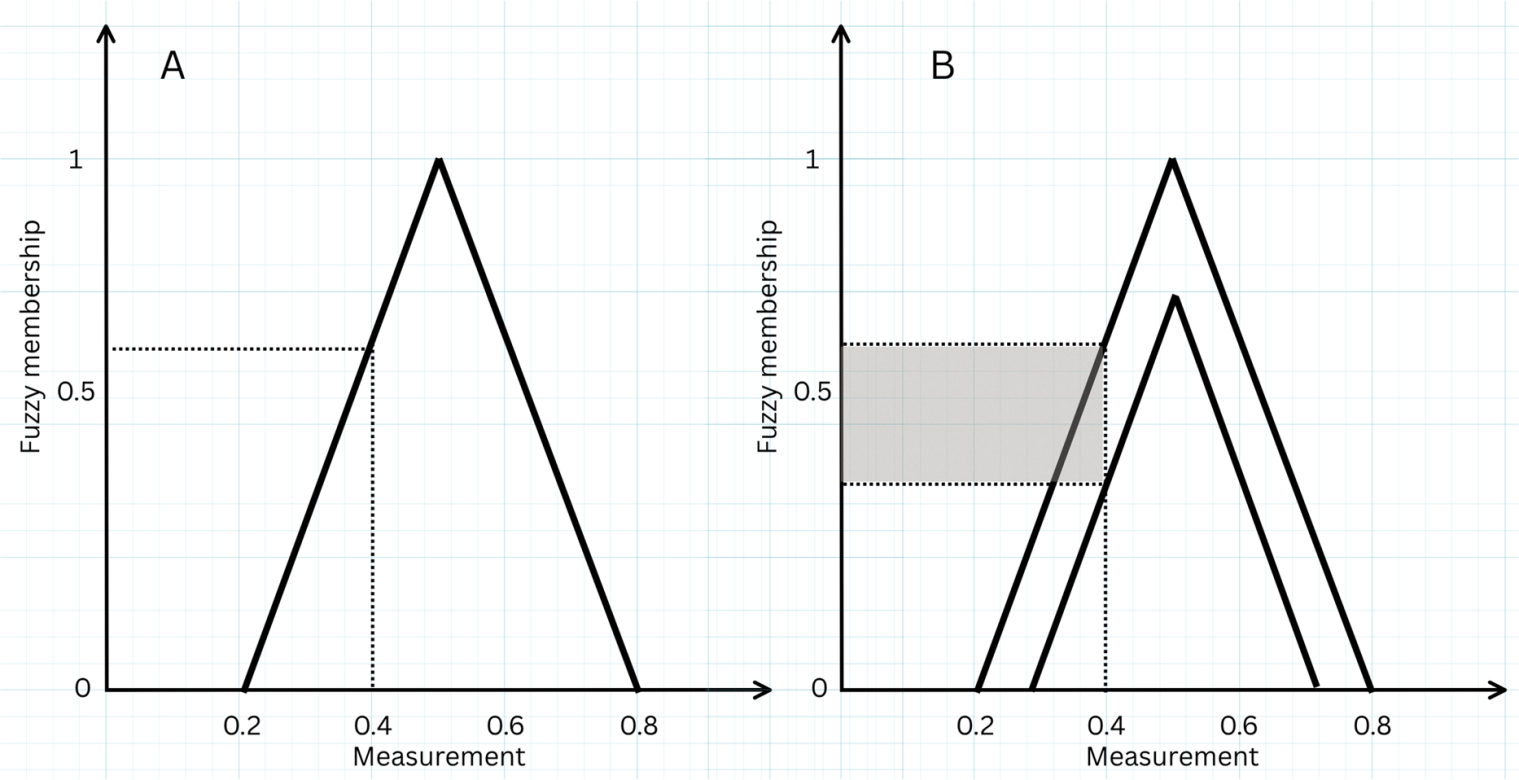
**A** Type 1 Fuzzy membership function where measurement values correspond to a single membership value compared to **B**: a type-2 Fuzzy membership function where, for a measurement value, a range of membership values are possible

A solution to the tension between Boolean and fuzzy frameworks lies in the modelling of higher-order vagueness, for example using a *type*-*2* fuzzy set, in which the membership function is also fuzzy, and hence an $\alpha$-cut returns a fuzzy (rather than Boolean) set (Zadeh 1975). Though there are a theoretically infinite number of higher-order fuzzy sets (*type-n* fuzzy sets), their impact decreases and mathematical complexity increases with each level, so models in applied research tend to be limited to *type-2* fuzzy sets (Fisher 2010). The practical advantage of a *type-2* approach is that it permits an understanding of characterisation uncertainty for a given location (i.e., two pixels classified the same, but understood to have different uncertainties associated with that classification). As a rudimentary example to illustrate the difference in nature between these approaches: in a Boolean approach, a given location might be described as “grassland”; in a *type-1* fuzzy approach as having a “grassland” membership of 0.6 and a “heather” membership of 0.4; and in a *type-2* fuzzy approach as having a 0.6 (± 0.15) membership of “grassland” and a 0.4 (± 0.2) membership of “heather” (Fig. 1) illustrates this principle.

However, the widespread uptake of fuzzy methods in landscape ecology (and geographical information science more generally) has been hampered by multiple challenges. The lack of successful integration of approaches based on fuzzy GIS techniques into landscape ecology methods has both technical and theoretical root causes. From a technical perspective, a major challenge stems from the need to determine a suitable function by which membership can be determined for a given location, which is either extremely difficult to determine or (more frequently) arbitrary (Ahlqvist et al. 2000). In the context of land cover, fuzzy classification is generally operationalized in the form of a fuzzy classification algorithm, such as a fuzzy $c$-means classifier (FCM). The challenge is that such functions are highly sensitive to their parameters, whilst the values for those parameters are rarely well justified (e.g., the arbitrary ‘fuzziness’ parameter in FCM). Approaches based on poorly justified functions, or classifiers with poorly-justified parameters provide little improvement over the Boolean classification schemes that they seek to replace. Huck et al. (2023), provide an alternative to the definition of an arbitrary membership function through Fuzzy Bayesian Inference (FBI), which generalizes probabilistic induction into a fuzzy context, providing an empirical approach to fuzzy membership that removes the need to define an arbitrary membership function (Huck et al. 2023). However, a limitation of FBI is the computationally intensive nature of Bayesian inference methods and the need for multiple sources of independent evidence, which presents a significant barrier to adoption at the landscape scale. Nevertheless, the broad concept behind FBI, whereby uncertainties in a learner-based classifier is used to determine membership, can be generalized and applied to any classification method capable of producing uncertainties, such as a Random Forest (RF) classifier.

From a theoretical perspective, fuzzy approaches to land cover classification have largely been explored within geographical information science where questions have centred on issues of classification. Hence, such studies have pushed theoretical boundaries concerned with geographical representation but neglected the relevance of such inquiry to ecological questions. For example, the value of model uncertainty was previously identified by Loosvelt et al. (2012), who used the mean probability as the basis of an estimate of uncertainty in the classification of SAR imagery. In the context of habitat delineation, these uncertainties around the membership of a pixel to each land cover class are operationally analogous to the determination of habitat transitions (ecoclines or ecotones). The authors however do not make this theoretical link. Whereas other studies have made this link (e.g. Arnot and Fisher 2007), they do not explore the ecological implications of leveraging uncertainty in landscape representation. As a result, the integration of uncertainty in land cover classification into key research areas of landscape ecology such as the nature and direction of influence of fragmentation on biodiversity, remains incomplete.

### Reconciling fuzzy approaches with aggregation-based methods

The representation of land cover in the form of *type-2* fuzzy membership provides a neat solution to the problem of representation, but can create operational challenges with respect to further analysis. For example, most landscape metrics are based on Boolean assumptions in which landscape objects (e.g., a contiguous region of woodland) can be partitioned from its surroundings. Approaches to analogous measures for fuzzy objects broadly constitute representing a single object as a series of nested boundaries determined by a series of $\alpha$-cuts (e.g., Zhan 1998; Fisher 2010; Dennis and Huck 2025). However, such methods are limited in their ability to model landscape processes and patch-landscape interactions because they do not reflect the tendency of habitat resources to aggregate in space (i.e. they do not delineate the patch). The development of methods that can operationalize fuzzy surfaces on land cover information within the process of habitat delineation remains an ongoing challenge. A potential solution to this is the use of Monte Carlo simulation for a random set of fuzzy habitat objects where the habitat value for each pixel is drawn from its respective distribution. Leveraging uncertainty to achieve a more comprehensive estimate of habitat has additional advantages. This is because each realization of habitat implies a correspondingly unique realization of distances between habitat patches, habitat-matrix transitions and landscape resistance. For example, instead of a single least-cost path, effective distances can be determined as the assessment of least cost paths between a set of fuzzy habitat objects. Here a fuzzy habitat object consists of groups of adjacent cells with membership to the habitat class above a given $\alpha$-cut. The ‘core’ region will be represented by the area in which fuzzy objects most consistently arise within a Monte Carlo simulation. The movement costs associated with each pixel can be derived according to weighted averages of cell membership to each class. Not only is this desirable but, functionally speaking, both object and distance delimitation should be seen as two sides of the same coin. That is to say, the distance between two objects is dependent on their assumed spatial boundaries. Hence, if objects are allowed to be fuzzy, so must the distances between them.

To date, several attempts have been made to develop methods that address the unlikely assumption that species always follow an optimal least cost route within the landscape by introducing uncertainty into effective distances between habitat patches through, for example, the use of random walks (Saerens et al. 2009), or circuit theory (McRae et al. 2008). We suggest two shortcomings of these approaches, however. Firstly, the random component is typically tuned using arbitrarily assigned values rather than being driven by the underlying data, and secondly, they do not offer a complete framework, ignoring the ‘random’ fluctuations that may also occur in the perception of habitat quality (in addition to perception of movement cost). We propose that a more comprehensive understanding of both habitat patches and their respective distances is possible through an approach that makes better use of the uncertainty inherent in landscape classification.

The purpose of this paper is to address the shortcomings described above through the presentation of a methodological framework that achieves a functional measure of habitat provision and navigates the continuity-contiguity impasse. This framework has two key components. First, we apply fuzzy set theory to leverage the uncertainty inherent in landscape classification methods and exploit this information through the use of Monte Carlo simulation. Secondly, we introduce the notion of functional habitat, a heuristic that captures the actual availability of habitat after accounting for species-specific requirements, the distribution of contributing resources and habitat-matrix interactions. Our work builds, therefore, on important precedents that operationalize vagueness in geographical representation (Fisher 2010; Arnot et al. 2004; Comber et al. 2010) and extends these concepts to an ecological context through their integration with species-specific spatial kernels and Monte Carlo propagation.

In this approach, we make a distinction between habitat area, habitat amount and functional habitat (Table 1). In the workflow that follows, we focus on vegetation cover types specifically, which are typically represented as Boolean classes in categorical maps. Although continuous, abiotic variables (e.g. moisture, temperature, light availability) undoubtedly contribute to habitat conditions, our interest is in the process and representation of fragmentation. Because research into habitat loss and fragmentation is overwhelmingly concerned with the removal of biodiversity-supporting land cover (primarily vegetation, Evju and Sverdrup- Thygeson 2016; Lindgren and Cousins 2017; Melo et al. 2017; Watling et al. 2020), we focus on land cover types in this study. Where abiotic and biotic conditions interact, such as when increasing soil moisture promotes wet woodland habitat, this can be delineated through a suitable land cover scheme with appropriate training data, or delineated in a continuous way when membership to multiple classes (e.g. water, woodland) is permitted (see Section “Achieving multivariate habitat delineation”). We note that datasets such as EUNIS (Chytrý et al. 2020) integrate multiple vegetation types and as such, offer an effective means of moving beyond single cover types in habitat definitions. Despite the availability of these valuable resources, in the context of fragmentation and patch dynamics, methods are still needed that better reflect space use by species that may not restrict themselves to the fixed boundaries that characterise Boolean habitat schemes.

**Table 1:** Habitat definitions used in this paper

Term	Definition
Habitat area	The spatial extent of cells classified as habitat in a Boolean (binary) scheme
Habitat amount	The sum of all cell values representing membership to the habitat class multiplied by the cell area
Multivariate Habitat	Habitat delineation that involves summing membership-weighted suitability values applied to all cover types thought to represent habitat resources
Functional habitat	Habitat amount based on membership values to all resources (cover types) that contribute to habitat, and accounting for (positive and negative) neighbourhood effects

This first of two papers details the theoretical foundation and methodological process underpinning our approach and supports the demonstration of a real-world application in a second paper (Dennis et al. this issue) based on a RF classification of a case study landscape. Subsequent sections present the core approach to habitat patch delineation. This is then extended to related estimates of between-patch distances before, finally, combining these into a graph-theoretic approach to modelling habitat connectivity. Although all ecological processes where patch delineation is required will be informed by our approach, we focus on connectivity as a natural exemplar given the key related parameters of patch size and isolation.

## Methodological approach

Conceptually, our approach bridges key theoretical positions in landscape ecology (the gradient and patch models of habitat) and geographical information science (geographical vagueness and image classification using type-1 and type-2 fuzzy membership). Operationally, we apply these concepts to the outputs of a classification algorithm. Here we take the example of a RF classifier though any classification algorithm from which uncertainty in the membership function can be extracted could be used. Briefly, RF is a learning algorithm comprising an ensemble of decision trees (i.e., a ‘forest’), each of which is trained on a bootstrapped sample of the training dataset before individually attempting to classify a location (Breiman 2001). When a pixel is classified, each tree returns a probability that the pixel is a member for each class, which is calculated as the proportion of samples from the training dataset in that ‘leaf’ (the end point of the decision tree) that were classified with the same class. As RF classifiers are normally implemented in a Boolean manner, the class with the greatest mean probability across all trees is then returned by the RF classifier as the selected class. This provides a pixel-level measure of classification uncertainty. Such models are typically then evaluated using a confusion matrix, which gives a corresponding class-level measure of classification uncertainty. These two uncertainty measures contain important complementary information (e.g., a pixel with a high confidence in a class that is generally poorly classified should have greater uncertainty than one with equal confidence but in a class that is a generally well classified), and so should be combined to give a more representative uncertainty value. The tree probabilities (the probability that a cell contains a particular class) can be updated based on the respective probabilities from the confusion matrix diagonal (i.e., the probability that a given class is correctly predicted by the model) using the power posterior method, which provides a straightforward means to address their non-independence (e.g., Friel and Pettitt 2008):

$$\alpha =1-\left|\rho \right|$$

1 $${P}_{c}=\frac{{P}_{1c}{\bullet { P}_{2c}}^{\alpha }}{{\sum }_{k=1}^{K}{P}_{1k}{\bullet { P}_{2k}}^{\alpha }}$$

where ${P}_{c}$ is the posterior probability of class $c$ being present in a given cell, ${P}_{1}$ and ${P}_{2}$ are probability vectors over $K$ classes (from the individual trees and the confusion matrix respectively) and $\left|\rho \right|$ is the absolute correlation value between the two vectors.

If we assume that the classifier is well-trained and performs well (which can be verified using standard metrics), then the composite classification probabilities described above reflect the uncertainties inherent in the land cover classification. Following the approach laid out in FBI (Huck et al. 2023), these uncertainties can then be taken as a fuzzy membership value for each pixel and class. Because the individual decision trees are trained using bootstrapped subsamples of the training dataset, the classification probabilities are not independent and so are considered as repeated estimates rather than as ‘new’ evidence, which is important as treating them as such would result in a substantial underestimation of the classification uncertainty. In the context of fuzzy set theory, the mean uncertainties for the RF therefore provide type-1 fuzzy membership values, whereas the individual uncertainties from each tree permit a type-2 fuzzy representation in which each membership value is represented as a fuzzy number (i.e., a distribution of possibilities, rather than a single value).

As in any landscape analysis, the successful execution of the method is predicated on the assumption that the classifier performs to an acceptable standard. However, whereas classifier performance is typically used to assess the correct assignment of pixels to their respective class, in our approach, we emphasize the opportunity afforded by a well-performing classifier to reflect properties of the landscape (e.g. gradients and variation caused by sub-pixel features). Next, we demonstrate the principle of a fuzzy habitat distribution using hypothetical landscapes in which the mean membership and associated uncertainty (i.e. the results of a classification algorithm) have been simulated.

### The example of a single patch

**Fig. 2:**
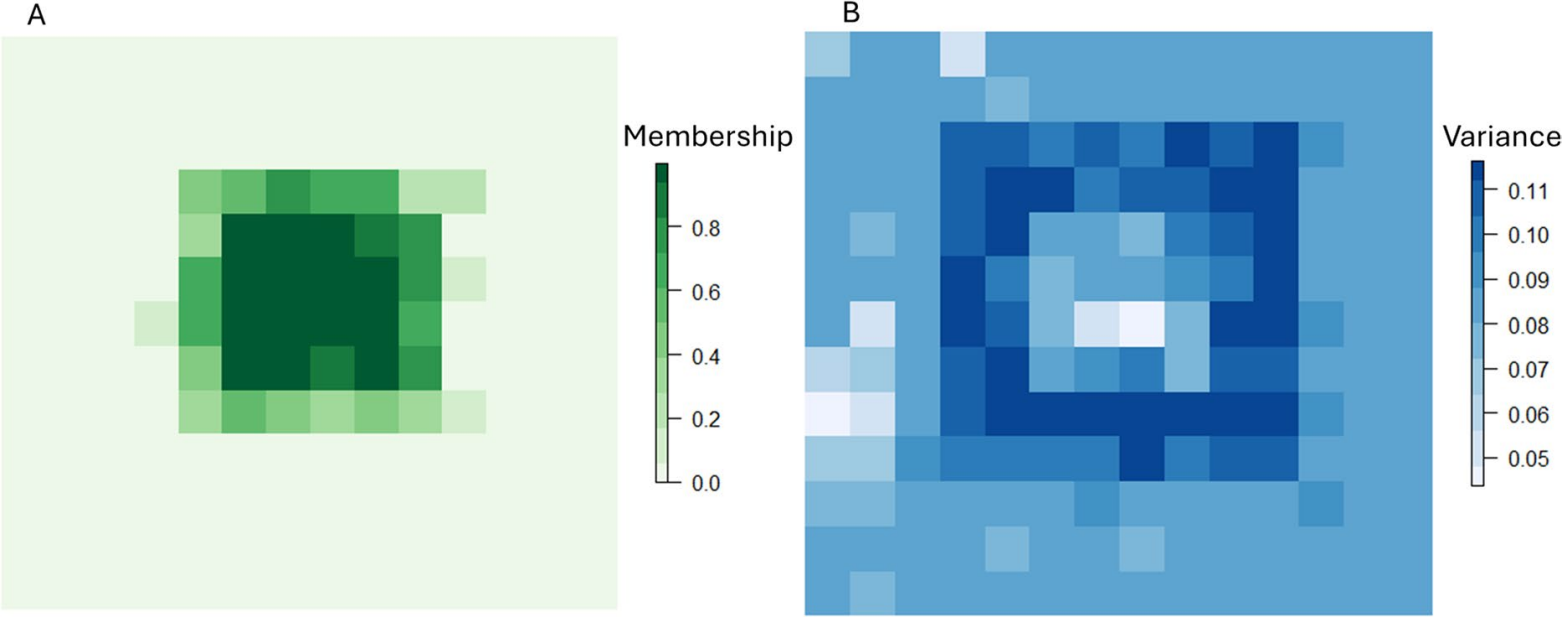
Example of **A** membership and **B** uncertainty (variance) for a low-contrast transition between habitat and the surrounding matrix. Values in A represent membership, analogous to the probabilities produced by a classifier (e.g. Random Forest) to habitat where we make, for illustrative purposes, the simplifying assumption that habitat consists of a single class (e.g. woodland). **B** represents the variance around these probabilities which can be obtained from the classifier

Figure 2 shows a simulated example of a low contrast transition between cells of high and low habitat membership, reflecting a situation in which ecotones exist between land covers. The key distinction, important for the development of the functional metrics that follow, is the high proportion of intermediate pixels in Fig. 2A where Fig. 2B represents the uncertainty (e.g. the variance associated with these values). In a typical image classification scenario, those pixels exhibiting a greater degree of membership uncertainty will generally occur at the transition between “core” land cover types and, hence, be assigned intermediate values.

A simple assessment of habitat can be made based on the minimum bounding polygon around adjacent cells that meet a certain threshold (e.g., an $\alpha$-cut of 0.5). A rudimentary calculation of habitat area would then be the geometric area of a polygon that contains adjacent cells with values above this threshold. A type-1 fuzzy assessment of habitat, on the other hand, would be the sum of the habitat membership values for all cells within the polygon (Eq. 2):

2 $$p{Hab}=\sum_{\mathrm{c=0}}^{\mathrm{|M|}}{M}_{c}$$

where $M$ is the set of habitat membership values for each cell contained within the bounding polygon $p$, $\mathrm{|M|}$ is the length of that set, and ${M}_{c}$ is the membership value of cell $c$ to the habitat class.

A measure of habitat *amount* would then be achieved simply by multiplying $p{Hab}$ by the cell area. This approach implies the removal of the requirement that patch size and habitat amount should be equivalent (as the cell area is weighted by its membership). Rejecting this assumption may seem counter to the intuition that the spatial extent of an area also reflects the “amount” that it contributes to the landscape. This idea, however, makes the unlikely assumption that habitat is evenly and completely distributed throughout that spatial extent. Whilst the *type-1* fuzzy assessment is a more faithful representation of the full information that we have on the landscape in comparison with a Boolean approach (i.e., it retains some of the uncertainty in the classification), it still ultimately assumes a single crisp boundary and a single membership value for each cell, and so is still subject to the shortcomings discussed above. A *type-2* fuzzy approach, however, permits a more comprehensive sense of the distribution of possibilities associated with habitat membership by retaining all of the information about the landscape (i.e., it retains all of the uncertainty in the classification). Practically, these uncertainties can be represented as a Beta distribution, which is a flexible distribution for continuous values in the range $[0, 1]$ and as such is widely used for the representation of possibility and probability values. A Beta distribution of possible membership values in each cell (one per class) in a surface then permits Monte Carlo simulation, whereby a large number of possible landscape scenarios can be randomly drawn from these distributions (i.e., with one value in each cell), allowing the uncertainty to be propagated throughout any analysis. Where patch delineation is required, an $\alpha$-cut can be applied to each landscape scenario. For the purposes of this manuscript, we have chosen $\alpha =0.5$, which provides an intuitive threshold (because cells with habitat membership above this value cannot exhibit a higher membership to another class), though any threshold can be set as informed by the user and landscape under investigation.

**Fig. 3:**
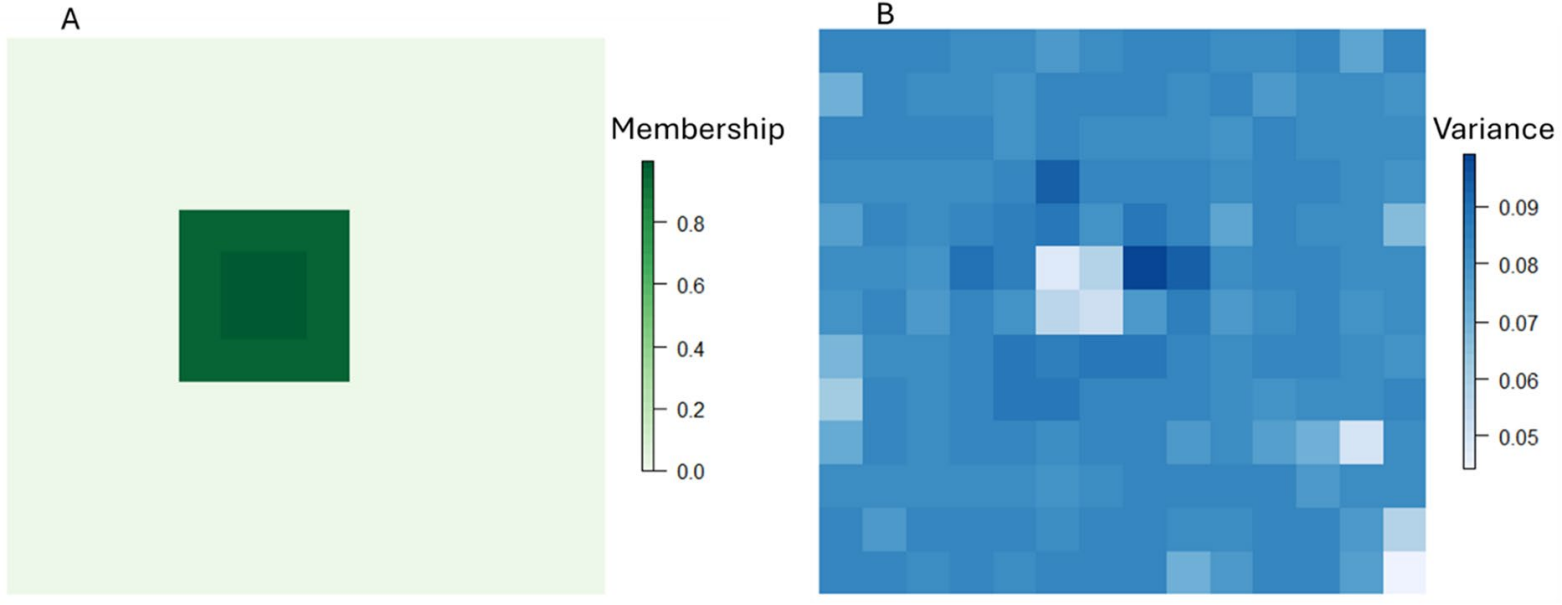
Example of **A** membership and **B** uncertainty (variance) for a high-contrast transition between habitat and the surrounding matrix. Values in A represent membership, analogous to the probabilities produced by a classifier (e.g. Random Forest) to habitat where we make, for illustrative purposes, the simplifying assumption that habitat consists of a single class (e.g. woodland). **B** represents the variance around these probabilities which can be obtained from the classifier

**Fig. 4:**
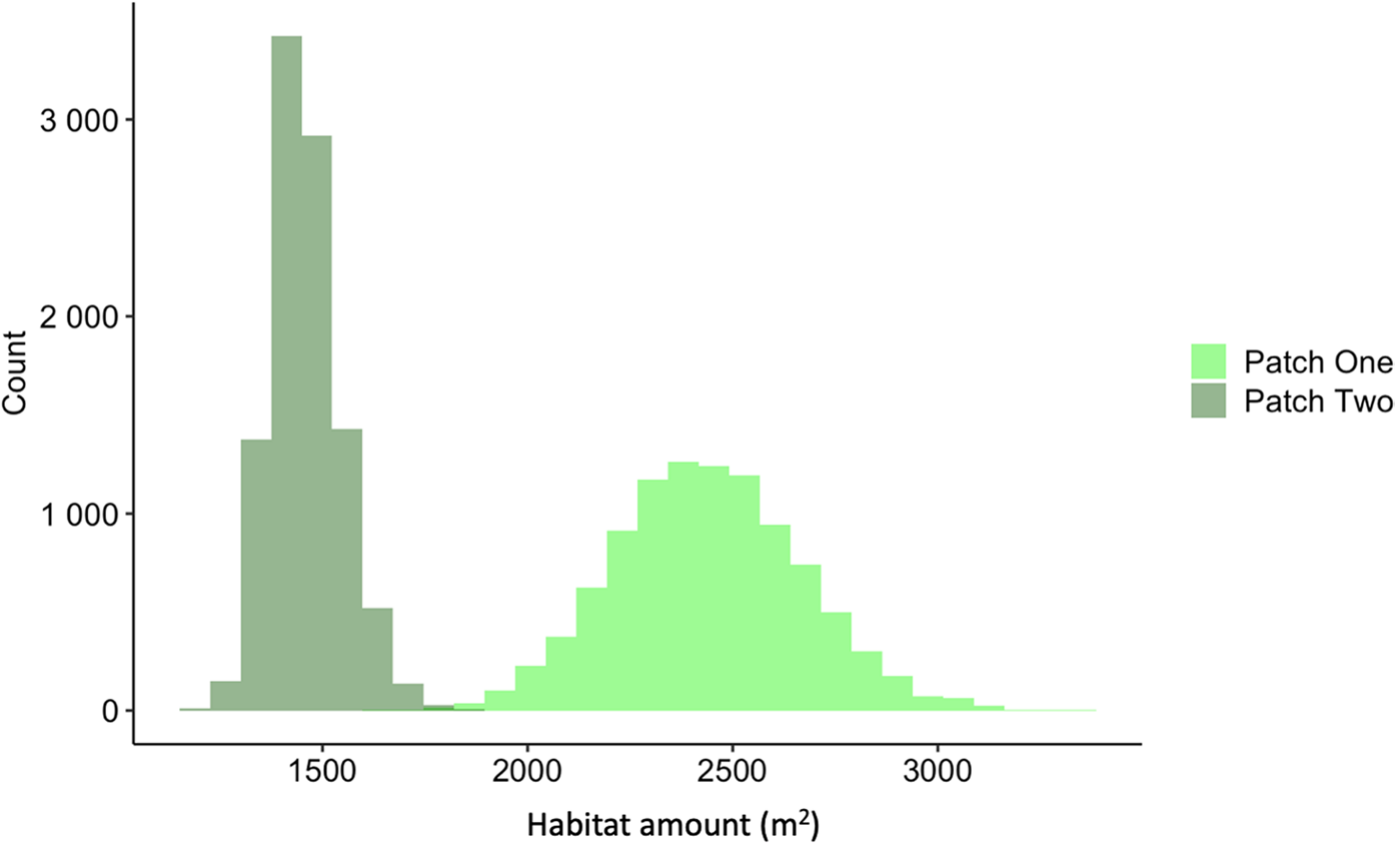
Histogram of values for 10,000 realizations of habitat amount for a low-contrast transition between habitat and the surrounding matrix (Patch One, where the distribution is based on the patch shown in Fig. 2) and for a high-contrast transition between habitat and the surrounding matrix (Patch Two, where the distribution is based on the patch shown in Fig. 3)

At each iteration of a Monte Carlo analysis, we can compute habitat values by removing all cells below the habitat membership threshold (i.e. 0.5) and applying Eq. 2. We repeat the process for 10,000 iterations for the patch shown in Fig. 2, multiplying $p{Hab}$ by the cell area to get our estimated distribution of habitat amount. The range of potential values for the metric can then be assessed using standard data visualization procedures such as a histogram (Fig. 4). We can compare this scenario to a high contrast example (Fig. 3) with fewer intermediate values between locations that exhibit high habitat membership and those with low membership. This might reflect a typical anthropogenic landscape such as when woodland is managed within specific land-use boundaries with “hard” edges formed with surrounding land-uses. Here, the contrast is much greater, resulting in a much smaller region of cells with high uncertainty.

Figure 4 compares the range of values computed for $p{Hab}$ for the low contrast scenario in Fig. 2 (Patch One: min = 1614; mean = 2440; max = 3354 m^2^) and Fig. 3 (Patch Two: min = 1130; mean = 1459; max = 1855 m^2^), reflecting the narrower distribution of values derived from the high-contrast scenario.

This presentation is a highly intuitive means of comparing the transition in space (e.g. edge versus core) of different habitat patches. A spatially continuous estimate of the core-to-edge transition can be achieved simply by computing, for each cell, the proportion of realizations in the Monte Carlo in which the cell meets the threshold ($\alpha$-cut). For example, for an interior woodland-dependent species, supposing an $\alpha$-cut of 0.5 is applied to delineate suitable habitat, those cells within the core woodland will naturally meet this threshold more consistently than cells towards the woodland edge. This can then be represented as a proportion (i.e. 0–1) in a continuous raster format. Note, however, that here we adopt a functional (species-oriented) definition of “core” and “edge” habitat such that, for a species dependent on woodland edge conditions, this would be “core” habitat where the woodland interior would represent the “edge” of habitat suitability. This is a key distinction and implies a shift from a geographically-derived measure of habitat (i.e. whereby edge is defined as the distance from the geographical centroid of a patch comprising a certain biotope) to a functionally-oriented measure based on the presence of suitable resources (i.e. multivariate habitat delineation).

Hence, the method allows for the delineation of (contiguous) patches at each iteration whilst permitting the representation of (continuous) habitat as a distribution. This is a key step in the reconciling of the *continuity-contiguity problem* as both contiguous and continuous perspectives are allowed without unnecessarily privileging either one. As a result, the procedure is directly transferable to any of the common area-, shape- or distance-based metrics relevant to fragmentation studies that require initial patch delineation whilst retaining information on more continuous qualities of habitat distribution.

### Estimating functional distances between patches

Next, we demonstrate how the application of a *type-2* fuzzy approach lends itself intuitively to another key process relevant to functional assessments of habitat availability: effective or functional distance. A common approach to modelling potential functional connectivity involves the calculation of distances between habitat patches. Typically, least cost paths are computed between patches, which are then incorporated into an assessment of connectivity based on patch size and the likelihood of a given species being able to traverse the least-cost distance (Dennis et al. 2024a). This process requires the cost of each cell (based on its cover type) to be estimated. The same method by which possibility of habitat membership can be represented as a distribution can be applied to any other land cover class occurring in the landscape. Hence, this means that the resistance value of any location in a landscape can be computed as a range of possibilities rather than as a single value. In addition, given that any cell can be allowed to reflect a degree of membership to each land cover class, a more comprehensive measure of the resistance value of each cell can be achieved by applying the membership values as weights. The cost value of each cell ($cost_{c}$) is then simply the sum of the products of the cell memberships to all classes and their corresponding resistance values:

3 $$c{ost}_{c} =\sum_{\mathrm{i=1}}^{\mathrm{|M|}}{ M}_{ci}{ R}_{i}$$

where: $M$ is the set of cell membership values to each land cover class for the current cell $c$, $\mathrm{|M|}$ is the length of that set, $R$ is the set of the corresponding resistance values, and ${M}_{ci}$ and ${R}_{i}$ are the cell membership and resistance values associated with land cover class $i$, respectively.

**Fig. 5:**
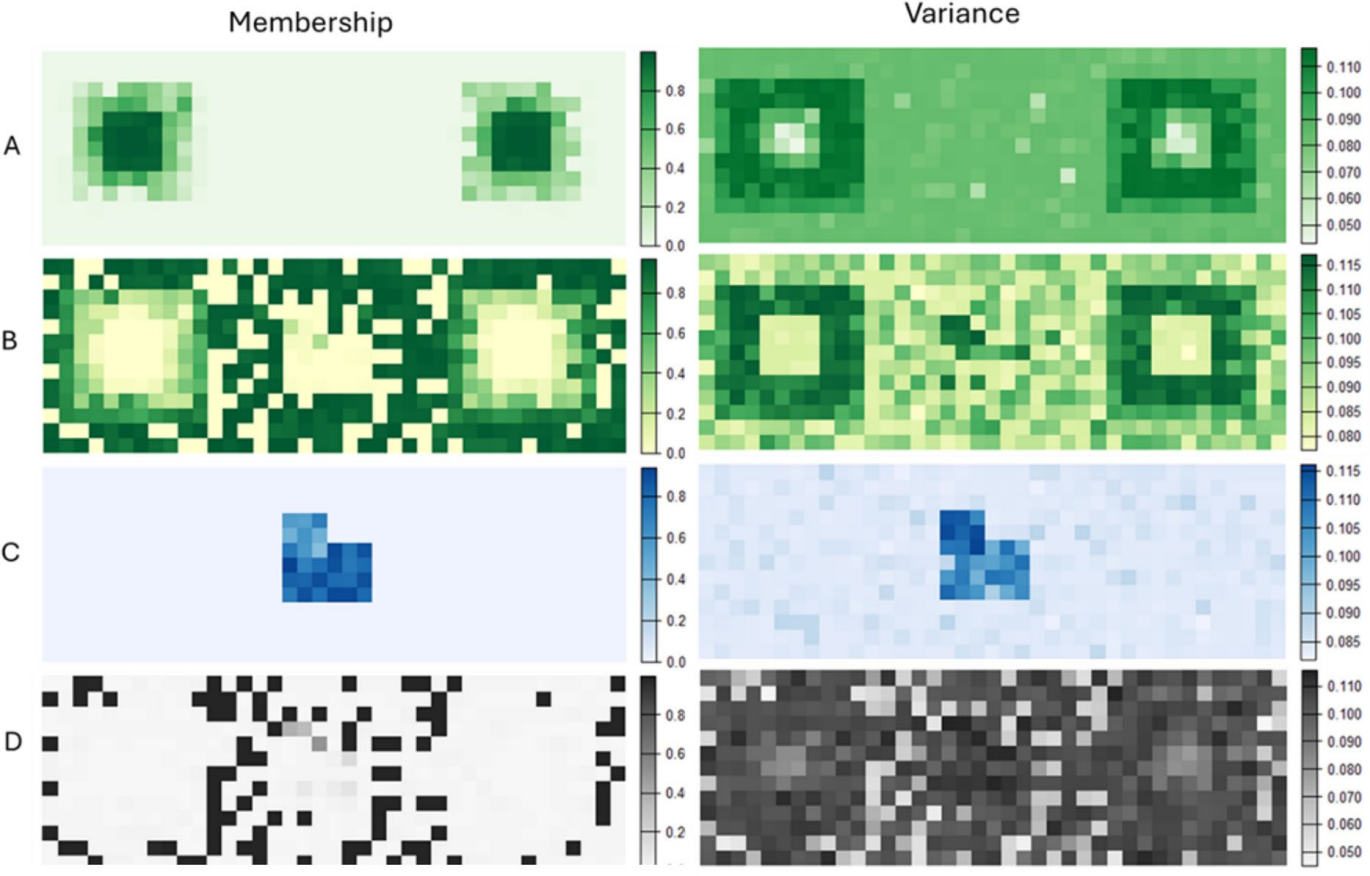
Membership and uncertainty for four land cover classes: **A** woodland, **B** grassland, **C** wetland and **D:** Urban. Here membership values correspond to the probabilities produced by a classifier (e.g. Random Forest) for each cover type. Right-hand surfaces for each cover type represent the variance around these probabilities, as can be obtained from the classifier

As above, a more comprehensive understanding can be achieved using Monte Carlo simulation, whereby the costs are calculated for e.g. 10,000 random landscapes drawn from the *type-2* fuzzy membership distributions. To demonstrate this idea, we generate four hypothetical surfaces, each representing cell membership to a different land cover class (Fig. 5). Accompanying each membership surface is a complementary uncertainty surface (variance in this example) from which, together, distributions can be drawn for Monte Carlo simulation.

**Fig. 6:**
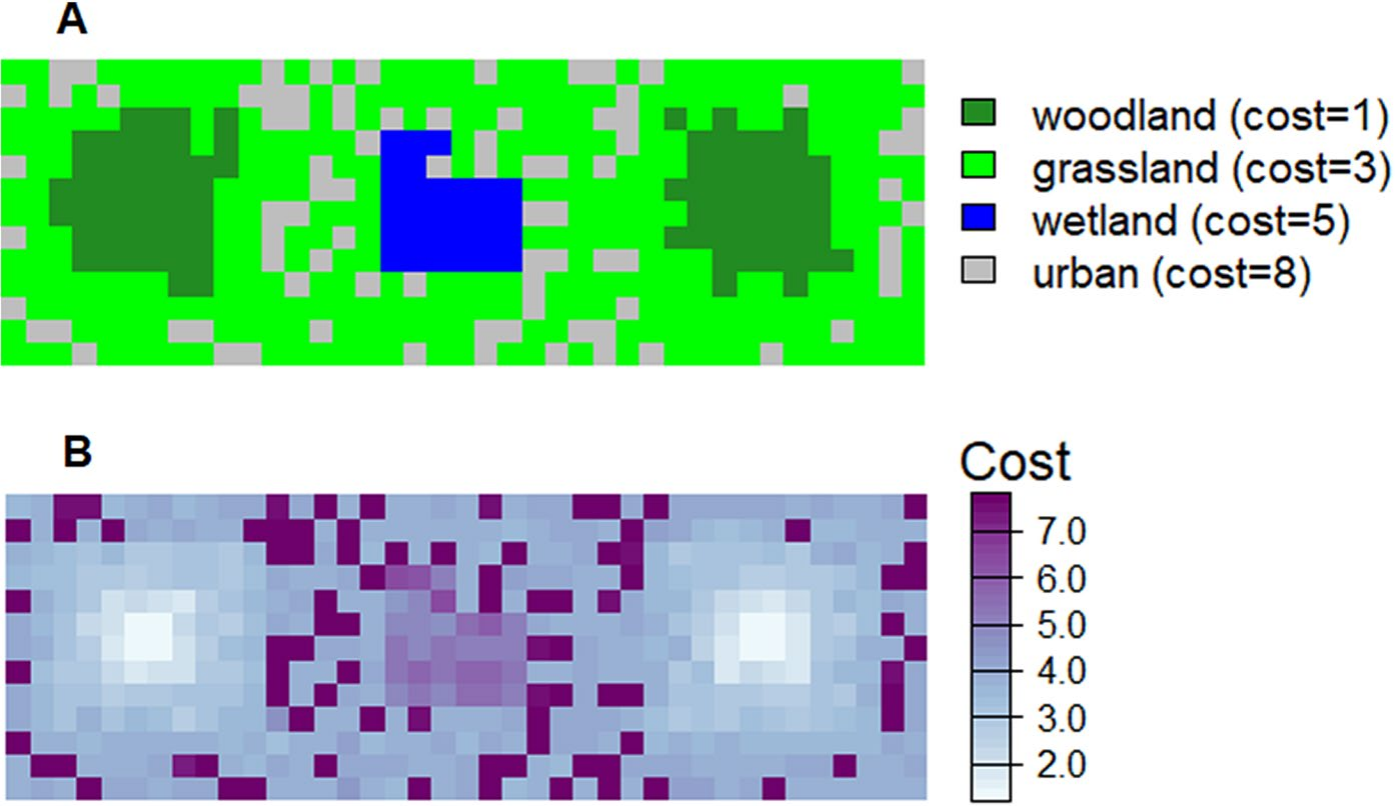
**A** Boolean representation of land cover and associated movement cost and **B** Movement cost for each cell as the sum of each class membership value multiplied by the cost associated with that class

Again, the greatest levels of uncertainty occur in cells with intermediate membership. Figure 6A gives the Boolean representation of the landscape where each cell belongs to only one class, that which exhibits the highest membership. Figure 6B gives the weighted cost surface based on *type-1* fuzzy membership values for land covers A–D in Fig. 5 (where we nominate woodland as the target land cover).

**Fig. 7:**
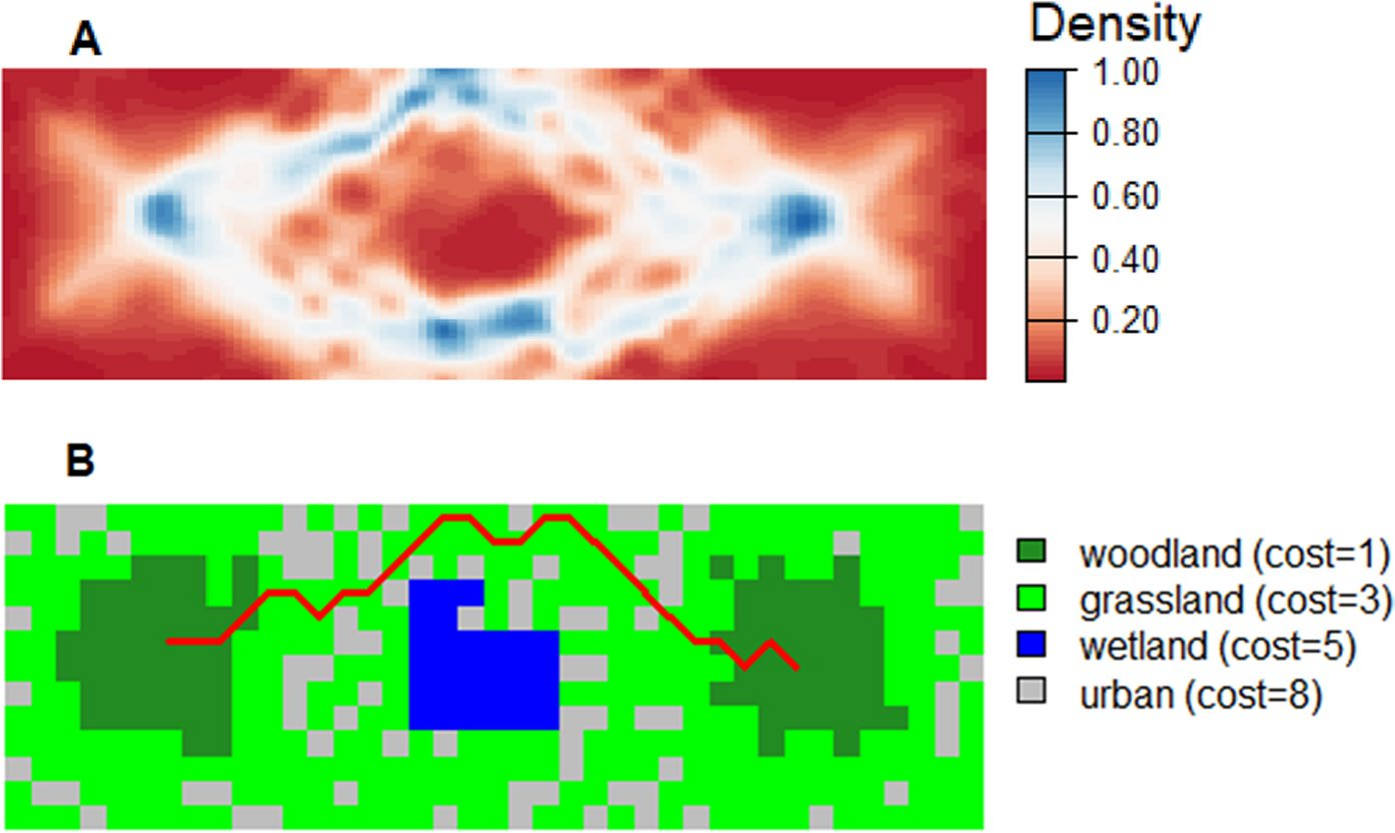
**A** Density surface (frequency with which each cell is located within the least cost path) based on the computation of 10,000 least cost paths between habitat patches where each computation is a realization of a Monte Carlo resampling process. **B** A single least-cost path based on a Boolean landscape

Figure 7A gives the density of possible least cost paths between the two habitat patches (the woodland class in Fig. 5A) for 10,000 random landscapes drawn from the *type-2* fuzzy membership distributions. For each landscape, resistance is determined as a function of the weighted cost calculated for each cell. The least-cost path is then calculated between the centroids of habitat patches consisting of adjacent cells whose membership to the woodland class exceeds the 0.5 $\alpha$-cut. Figure 7A was produced by applying the *density.psp* function from the *Spatstat* package (Baddeley et al. 2015) to polylines representing all least cost paths. Figure 7B gives the single optimal least cost path based on the Boolean representation of the landscape for comparison.

This approach to estimating effective distance has several advantages to previously published methods. Least-cost path analysis, though popular, has been criticized for its implied assumption that target species will have knowledge of, and take, the optimal least-cost path (Sawyer et al. 2011). For our landscape, the optimal path is shown in Fig. 7B and suggests that a modelled organism would always travel to the north of the central area of wetland. The fuzzy approach (Fig. 7A) suggests that paths to the south may also be taken with relatively high frequency. The assertion of an optimal least-cost path is itself predicated on the assumption that a single realization (membership) of cells to one or other land cover is realistic. To some degree, these unsatisfactory assumptions have been addressed by approaches that attempt to incorporate an element of randomness into path delineation. These include Circuit Theory (McRae et al. 2008) and random walks (Saerens et al. 2009), both of which seek to introduce redundancy into path modelling. As discussed above, however, a limitation of these approaches is that they are often tuned through arbitrary parameters and do not account for landscape classification uncertainty. Our approach does not require parameterisation beyond the assigning of initial cost values (as for any resistance-based distance measure). Rather, the “randomness” of the paths between patches (as in Fig. 7A) is determined as a function of uncertainty in the land cover classification. As such, the cells with highest density in Fig. 7A reflect the most common outcomes based on the information available on the landscape, with more redundant, but still possible, paths showing lower density.

### Achieving multivariate habitat delineation

The need to operationalize a multivariate *definition* of habitat demands the multivariate *delineation* of habitat in space. In this sense, a functional treatment of habitat is necessarily a spatial-ecological problem because the elements that determine habitat suitability (e.g. biological resources, physical structures, abiotic conditions) are themselves spatially structured and available to a given organism as a function of its use of space (e.g., as determined by its characteristic foraging or dispersal distance). Therefore, coherent and appropriate methods are required to bridge the gap where existing approaches fail to operationalize functional definitions of habitat.

**Fig. 8:**
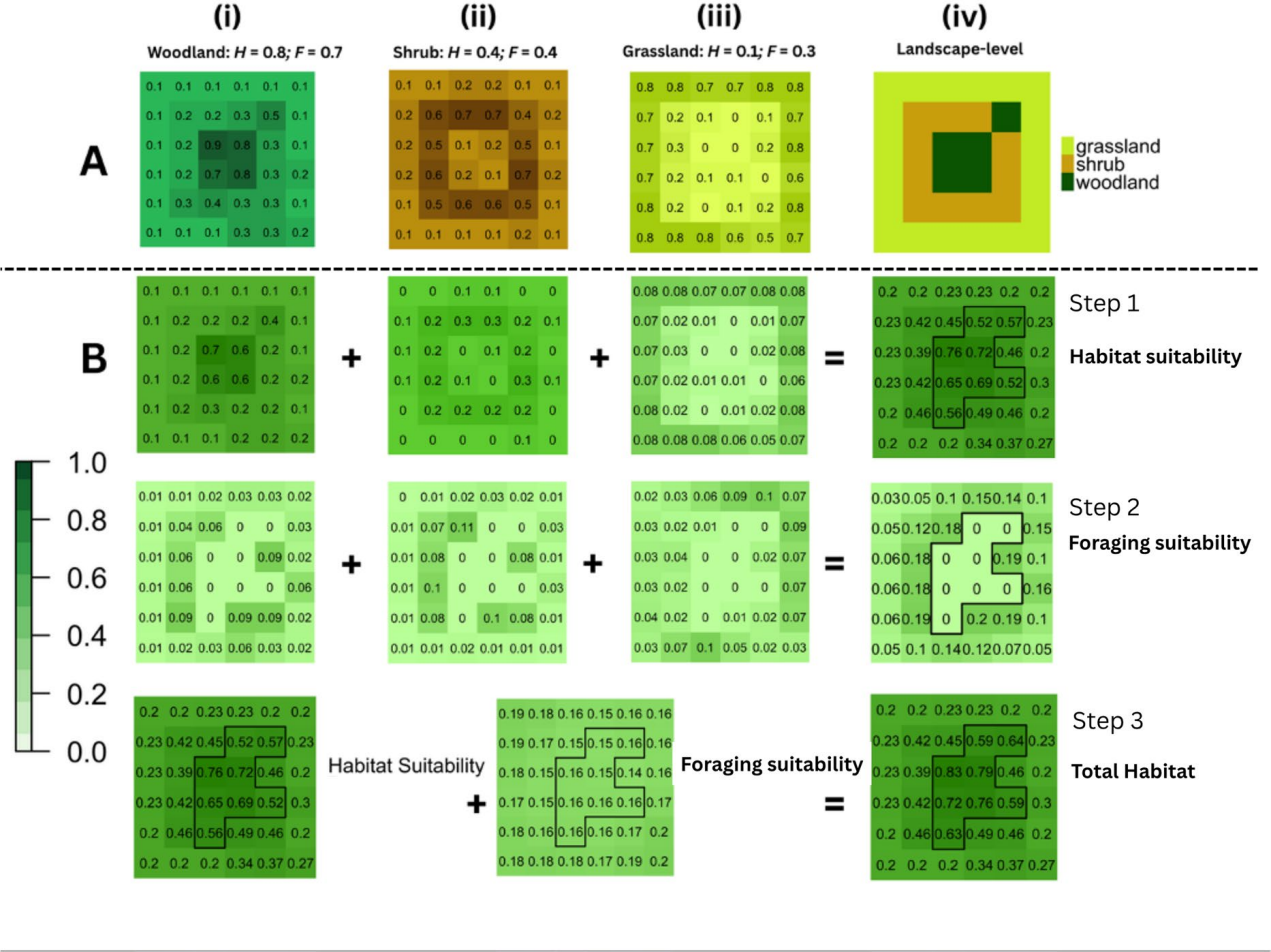
Example of steps leading to multivariate habitat delineation. 8A represents land cover class membership where 8B shows Step One, contribution to habitat, Step Two: foraging suitability, and Step Three represents the final calculation of habitat from suitability and complementarity. *H* denotes habitat suitability and *F* denotes foraging suitability. The colour ramp applies to all values in Steps 1 to 3 where darker colours represent greater contribution to total habitat. Note that, in this example, habitat suitability provides the primary contribution here to total habitat, with foraging suitability contributing secondary resources (and hence relatively lower contributions to total habitat). Whilst we expect this would be the usual case, this scheme can be adapted (i.e. through alternative weightings) according to species requirements and life-history traits

This can be done by leveraging type 1 uncertainty and spatial kernels in combination with species-specific resource-use parameters. Though true habitat specialists exist that rely on a particular biotope (e.g., oak woodland specialists), in which case a Boolean output may suffice if neighbourhood effects are thought to be negligible, many species have associations with multiple cover types (i.e. resources) which can be obligate (e.g., for the completion of the life cycle), complementary (e.g., representing supplementary forage outside the primary resource) or negative (e.g. due to inputs from adjacent land use). This has two major implications for the definition and subsequent delineation of suitable habitat. Firstly, it suggests that locations in space may contribute to habitat suitability as a function of the degree to which they represent one or more resources (which means that intermediate positions between classes provide useful information). Secondly, the degree to which a location in space contributes to habitat suitability is not only a function of the cover type at that location but also of those in nearby locations that occur within a species-relevant distance (i.e. such as that reflecting its home range or characteristic foraging distance). We propose that there is a logical parallel between the estimation of contributing nearby resources and the importance of nearby patches in meta-populations models. In the case of the latter, the size (as a surrogate for quality) and isolation of patches typically determine their contribution to the potential functional connectivity of the landscape (Moilanen and Hanski 2001). In the case of resource availability in the local environment, we invoke the same logic, but with two key modifications. Firstly, we do not assume that patch area (size) is a useful proxy for habitat amount and, secondly, we focus on estimating habitat provision as the cumulative contribution of all suitable resource (cover) types (rather than a single biotope) within an appropriate distance of a location in space. Here we make a clear distinction between suitability values related to habitat and foraging where the former primarily represents opportunities for nesting and breeding, and the latter represents secondary, complementary resources. Our definition of functional habitat therefore formally incorporates the notion of resource complementarity into habitat delineation. Figure 8 gives a hypothetical example of this process (see Sect. “Parameterising multiple resource types into a multivariate delineation of habitat and effective distances” for full details of how positive and negative neighbourhood effects can be parameterized and combined for habitat estimation). Figure 8A gives the membership of each cell in the example landscape to woodland (i), shrub (ii) and grassland (iii) cover types with the Boolean classification given in column (iv). Figure 8B describes the process of assigning the contribution of each cover type to habitat (Step One) as the product of membership of each cell and the corresponding habitat suitability weights (*H*) assigned to each type (where *H* can be determined through expert consultation, literature search or empirically with available data). Step Two involves assigning to each cell a foraging potential value, determined as a function of the foraging suitability (*F*) of each class, the cell membership values and the distance to neighbouring habitat cells (where habitat cells are those with habitat suitability equal to or greater than 0.5 in Step One). Finally (Step Three), total habitat is the sum of habitat suitability and complementarity where the latter is total foraging potential within an appropriate distance (e.g. reflecting the species’ home range) of each cell.

The idea of a resource-based definition of habitat that includes multiple, spatially disjoint biotopes is not new to landscape ecology, but is present in previous attempts to unlock the conundrum of habitat as an emergent property of species-landscape interactions (Dennis et al. 2007; 2014; Hartemink et al. 2015; Turlure et al. 2019). The notion that the amount of habitat within an “appropriate” distance is sufficient to determine species richness points to the influence of neighbourhood effects with respect to habitat provision and is a prominent feature of the habitat amount hypothesis (Fahrig 2013; Watling et al. 2020). However, this assertion does not move us closer to a functional definition of habitat as long as studies continue to be based on single biotopes (e.g. Evju and Sverdrup- Thygeson 2016; Lindgren and Cousins 2017; Melo et al. 2017; Watling et al. 2020), with the “appropriate distance” based on distances selected through model performance rather than on an understanding of species-specific parameters. A fundamental motivation of our approach is the observation that, if delineation is predicated on a binary view of habitat versus non-habitat then, by extension, fragmentation is likewise underpinned by a structural (geometric) rather than functional perspective. It follows that fragmentation-biodiversity studies may be undermined by an inappropriate definition of fragmentation itself. This highlights the need for a framework capable of integrating theoretical and operational aspects of habitat determination.

### Parameterizing multiple resource types into a multivariate delineation of habitat and effective distances

Next, we demonstrate in detail how to parameterize the contribution of multiple land cover types into a final measure of functional habitat incorporating positive and negative neighbourhood effects. We suggest two key steps to carrying out a multivariate delineation of habitat. First, the habitat value for each cell in the landscape is determined as the weighted sum of the memberships to all land cover types where the weights reflect each type’s contribution to habitat according to the logic set out in Fig. 8. This first step is carried out according to:

4 $$habitat_{c}=\sum_{\mathrm{i=1}}^{\mathrm{|M|}}{ M}_{ci}{ H}_{i}$$

where: $M$ is the set of membership values to each land cover class $i$ for the current cell, $\mathrm{|M|}$ is the length of that set, $H$ is the set of the corresponding habitat suitability values for each land cover class, and ${M}_{ci}$ and ${H}_{i}$ are the cell membership and habitat suitability values, respectively, associated with class $i$. Once *habitat*_c_ (range: 0–1) is computed for each cell, an $\alpha$-cut of 0.5 is used to determine habitat from non-habitat cells and individual patches delineated as groups of adjacent cells using a queen neighbourhood rule (eight nearest neighbours).

**Fig. 9:**
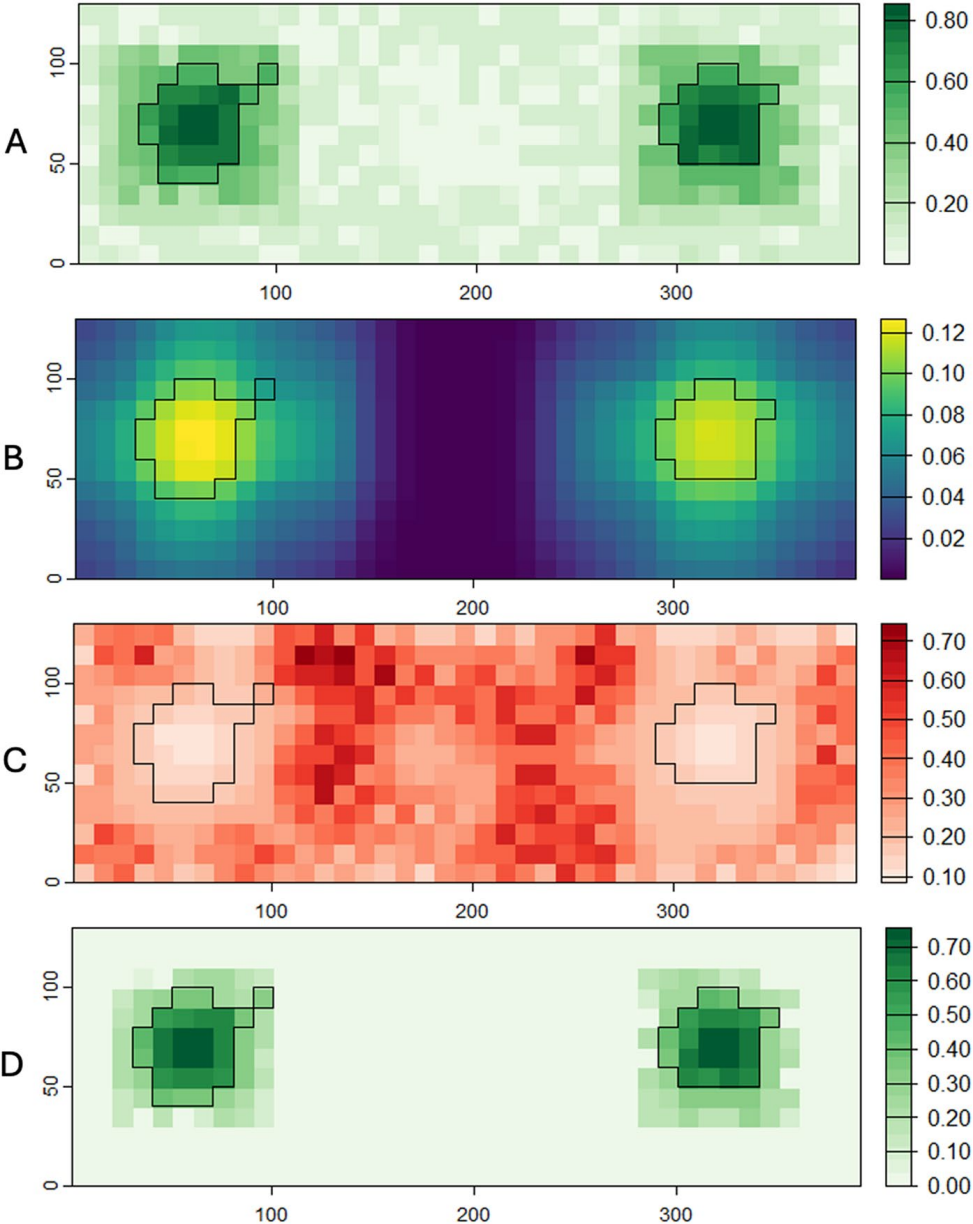
Habitat delineation based on the contribution of all cover types to habitat suitability and positive/negative neighbourhood effects.** A** Weighted habitat suitability; **B** ${nPos}_{c}$ computed for all cells in the landscape; **C** Negative neighbourhood effect surface associated with urban land cover; **D** Final functional habitat estimate considering the contribution of all land covers to habitat suitability and foraging potential, and negative neighbourhood effects. Habitat boundaries are shown for cells exhibiting ${fHab}_{c}$ ≥ 0.5

For our example landscape (Fig. 5), assigning habitat suitability values of 0.9, 0.1, 0, and 0 to woodland, grassland, wetland and urban land covers, respectively, gives an estimate of habitat shown in Fig. 9A. In the second step, estimates of negative and positive neighbourhood effects on habitat cells within each patch are determined using the *type-1* membership of neighbouring cells within a) an assumed edge effect distance exerted by deleterious land cover types and b) the foraging distance, respectively, of cells exhibiting ${habitat}_{c}$ values above the $\alpha$-cut.

#### Modelling negative neighbourhood effects

The modelling of negative neighbourhood effects can be achieved by exploiting *type-1* fuzzy membership. Land cover types that are detrimental to habitat quality exert an effect at a distance thought to be specific to each cover type (Watts and Handley 2010). To capture this process, we make use of a negative exponential kernel whereby the strength of the neighbourhood effect from each cell is a function of the membership of that cell to the deleterious land use and the distance from that cell (Dennis et al. 2024a). Based on this approach, Dennis et al. (2024b) previously demonstrated that the variable influence of connectivity on woodland mammal richness was attributable to negative neighbourhood effects associated with urban land cover.

For both negative and positive neighbourhood effects, we refer to the likelihood that neighbouring cell *k* has an effect on current cell $c$ as $P_{ck}$:

5 $${P}_{ck} ={(e}^{-\alpha {D}_{ck}})$$

where *e* is the natural exponent, ${D}_{ck}$ is the distance between cell *c* and neighbouring cell *k*, and $\alpha$ is a constant that determines the likely neighbourhood effect at distance ${D}_{ck}$. For negative neighbourhood effects, $\alpha$ is set such that ${P}_{ck}$ is 0.01 at the maximum Euclidean distance for which a given land cover is assumed to exert an effect, according to:

6 $$\alpha =\frac{-\mathrm{log}(d)}{maxD}$$

where $maxD$ is parameterized as the maximum edge effect distance, log is the natural logarithm and $d$ is a distance decay parameter which we set to 0.01 reflecting the strength of effect at $maxD$.

In both stages of the multivariate habitat delineation, key input parameters (i.e. for habitat suitability values and maximum edge effect distance) need to be estimated. Though we use arbitrary values for demonstration purposes here, in practice such values can be determined through expert consultation or through a literature search (see Dennis et al. this issue, for an example). Note that such values also imply a degree of uncertainty related to the confidence with which they are reported. These uncertainties can easily be incorporated into our approach by representing them as random variables in the Monte Carlo Analysis. Equally, these values can be derived empirically, such as through a species distribution modelling framework. Whilst operationally more feasible, this presents its own challenges. For example, where habitat associations are sought, the use of Boolean classified images (e.g. Giachello et al. 2025) does not escape the issues set out above in relation to the *continuity-contiguity problem*. Similarly, estimates based on unclassified optical imagery (e.g. Halstead et al. 2019) are purely correlative, with no mechanistic basis. As such, values derived in this way cannot be assumed to be transferable, nor do they contribute to a theoretically supported definition of habitat (Dennis et al. 2007; 2014; Hartemink et al. 2015; Turlure et al. 2019). In addition, the computation of functional distances between resources is either unreliable (in the Boolean case) or impossible (in the case of unclassified imagery). However, note that, if associations are based instead on *type-1* membership to each cover type and modelled through *type-2* fuzzy classification, SDMs and other modelling approaches that take habitat patches as inputs can be combined with our approach, thus avoiding the *continuity-contiguity problem*.

The negative neighbourhood effect ${nNeg}_{c}$ exerted on each habitat cell $c$ is then:

7 $${nNeg}_{c}=\sum_{\mathrm{k=1}}^{|K|} \sum_{\mathrm{i=1}}^{{|M_{k}|}}{P}_{ck}{ M}_{ki}$$

where $K$ is the set of cells in the neighbourhood of cell *c* assumed to exert an edge effect, *M*_k_ is the set of land cover class membership values for neighbouring cell  $k$, $P_{ck}$ is the likelihood of neighbouring cell *k* exerting a neighbourhood effect on cell *c*, and ${M}_{ki}$ is the membership value to land cover class $i$ in neighbouring cell $k$ as per Eq. 5.

Applying this process to the urban layer from Fig. 5D (assuming only the urban class exerts a negative effect) and assuming that the negative neighbourhood effect of a cell with an urban membership of 1 exerts an edge effect up to a maximum of 5 cells, gives the “neighbourhood effect” surface depicted in Fig. 9C. The computation of negative neighbourhood effects can equally be applied to cost as well as to habitat surfaces, such that cost increases with proximity to urban land use (in our example) and functional movement cost for each cell then becomes:

8 $${functionalCost}_{c}=\sum_{\mathrm{i=1}}^{{|M|}}{ M}_{ci}{ R}_{i} + \sum_{\mathrm{k=1}}^{{|K|}}\sum_{\mathrm{i=1}}^{{|M_{k}|}} {P}_{ck}{ { M}_{ki}R}_{i}$$

where $M$ is the set of cell membership values to each land cover class for the current cell *c*, $R$ is the set of corresponding resistance values, ${M}_{ci}$ and ${R}_{i}$ are the cell membership and resistance values, respectively, associated with land cover class $i$, *K* is the set of cells in the neighbourhood of cell *c* assumed to exert a neighbourhood effect, *M*_k_ is the set of land cover class membership values for neighbouring cell *k*,  ${P}_{ck}$ is the likelihood of cell *k* exerting a neighbourhood effect on cell *c* (as per Eq. 5), and *M*_ki_ is the membership value of neighbouring cell *k* to land cover class *i*.

This reflects the mechanistic understanding that nearby deleterious land uses may affect movement costs and avoids unsupportable situations such as where a line of street trees in an urban area exhibits the same movement cost as a tree line intersecting grassland or scrub.

#### Positive neighbourhood effects

We model positive neighbourhood effects as the potential contribution of neighbouring land covers to habitat quality. We take the example of a species that is able to exploit resources within a characteristic foraging distance. In such a case we model the contribution of each cell within this distance to habitat as a function of foraging suitability ${(forage}_{c}).$

9 $${forage}_{c}=\sum_{\mathrm{i=1}}^{\mathrm{|M|}}{ M}_{ci}{ F}_{i}$$

where $M$ is the set of cell membership values to each land cover class for the current cell $c$, $\mathrm{|M|}$ is the length of that set, $F$ is the set of the corresponding foraging suitability values, and ${M}_{ci}$ and ${F}_{i}$ are the cell membership and foraging suitability values, respectively, associated with landcover class $i$. The likelihood of secondary resources being accessible from habitat patches is then parameterised as ${P}_{ck}$ but where ${D}_{ck}$ is modelled as the functional cost distance betweenhabitat and neighbouring cells and α is set such that ${P}_{ck}$ is 0.05 at the maximum estimated foraging distance (set to 100 m in our example). This is achieved by setting $maxD$ as the maximum foraging distance and $d$ to 0.05 reflecting the probability of movement to a cell at $maxD$.

The total amount of complementary resources available to each habitat cell $c$ $({nPos}_{c}$) is then the sum of the product of ${P}_{ck}$ and foraging suitability values for all neighbouring cells *k* within distance $maxD$, normalized by the total number of cells:

10 $${nPos}_{c}=\frac{\sum_{k=1}^{|K|}\sum_{i=1}^{|M_{k}|} {P}_{ck} {M}_{ki}{F}_{i}}{|C|}$$

where *C* is the set of cells within distance $maxD$ of habitat cell $c$, *K* is the set of cells in the neighbourhood of cell *c* assumed to exert a neighbourhood effect, *M*_k_ is the set of land cover class membership values for neighbouring cell *k*, *P*_ck_ is the likelihood of neighbouring cell *k* exerting a neighbourhood effect on cell *c* (parameterised by measuring *D*_ck_ as functional cost), and *M*_ki_ and *F*_i_ are the membership of neighbouring cell *k* and foraging suitability value, respectively, associated with land cover class* i*.

Assigning foraging suitability values of 0.8, 0.5, 0.3 and 0.2 to woodland, grassland, wetland and urban land covers, respectively, yields the surface of ${nPos}_{c}$ values in Fig. 9B. The final estimate of functional habitat ${fHab}_{c}$ (Fig. 9D) is then obtained as:

11 $${fHab}_{c}={habitat}_{c}+{nPos}_{c}-{nNeg}_{c}$$

### Modelling an ecological process

The functional measures obtained through the previous steps can be combined to estimate fragmentation-related outcomes. Here we focus on the development of a potential functional connectivity metric that incorporates uncertainty in the input parameters and the estimation of functional habitat. This is achieved using a derivation of Hanski’s Incidence Function (Hanski 1998) and applies the Monte Carlo approach described above to computing the key components of habitat amount and distance. Specifically, we update the Edge-weighted Habitat Index (EHI) as proposed in Dennis et al. (2024a) through the application of our functional habitat delineation approach, incorporating a multivariate habitat definition and neighbourhood effects. The involves the computation of an instance of ${fHab}_{c}$ at each iteration with the resulting functional habitat patches (equivalent to *pHab* in Eq. 2 but where *M*_c_ is replaced by *fHab*_c_) entered into the modified EHI metric. We refer to this as the reachable functional habitat (RFH) according to:

12 $$RFH= \frac{\sqrt{{\sum }_{p=1 }^{n}{\sum }_{q=1 }^{n}{ pHab}_{p}{{A} pHab}_{q}{A}{ P}_{pq}^{*}}}{{A}_{L}} \times 100$$

where: ${pHab}_{p}$ and ${pHab}_{q}$ are the functional habitat values for patches $p$ and $q$, ${A}$ is the cell area, ${A}_{L}$ is the total landscape (i.e. study extent) area and dispersal probability between patches $p$ and $q$ is defined as the maximum probability of movement (where ${P}_{pq}^{*}$ is the maximum product probability of all the possible paths between patches $p$ and $q$) based on shortest paths in a probabilistic patch-based graph. The result is multiplied by 100 to render the metric as a percentage. 

**Fig. 10:**
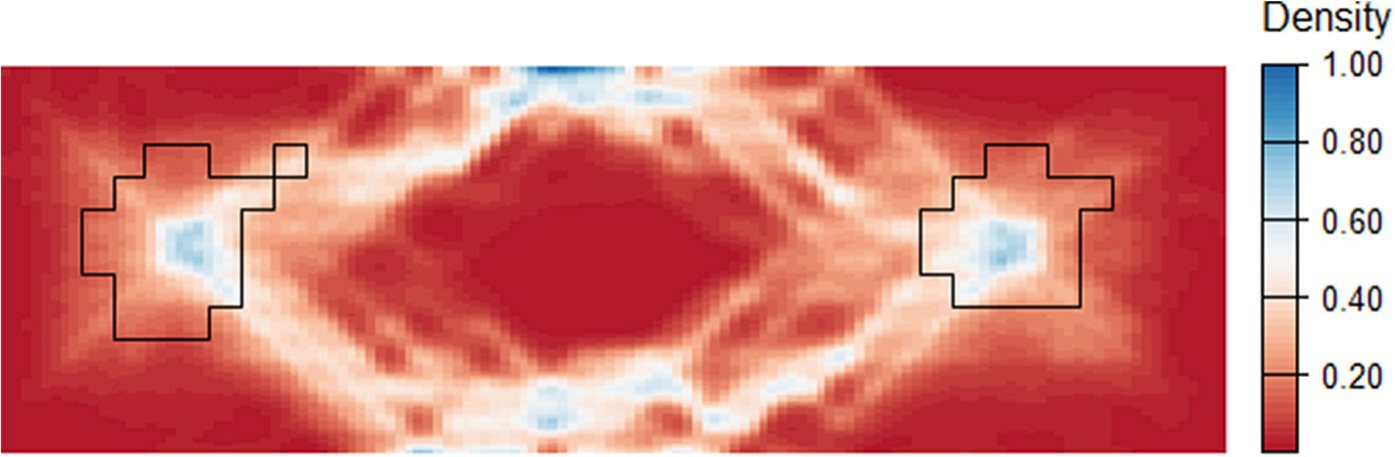
Least cost path density for 10,000 iterations of the Reachable Functional Habitat computation. Habitat boundaries (in black) are shown for contiguous cells exhibiting ${fHab}_{c}$ ≥ 0.5

**Fig. 11:**
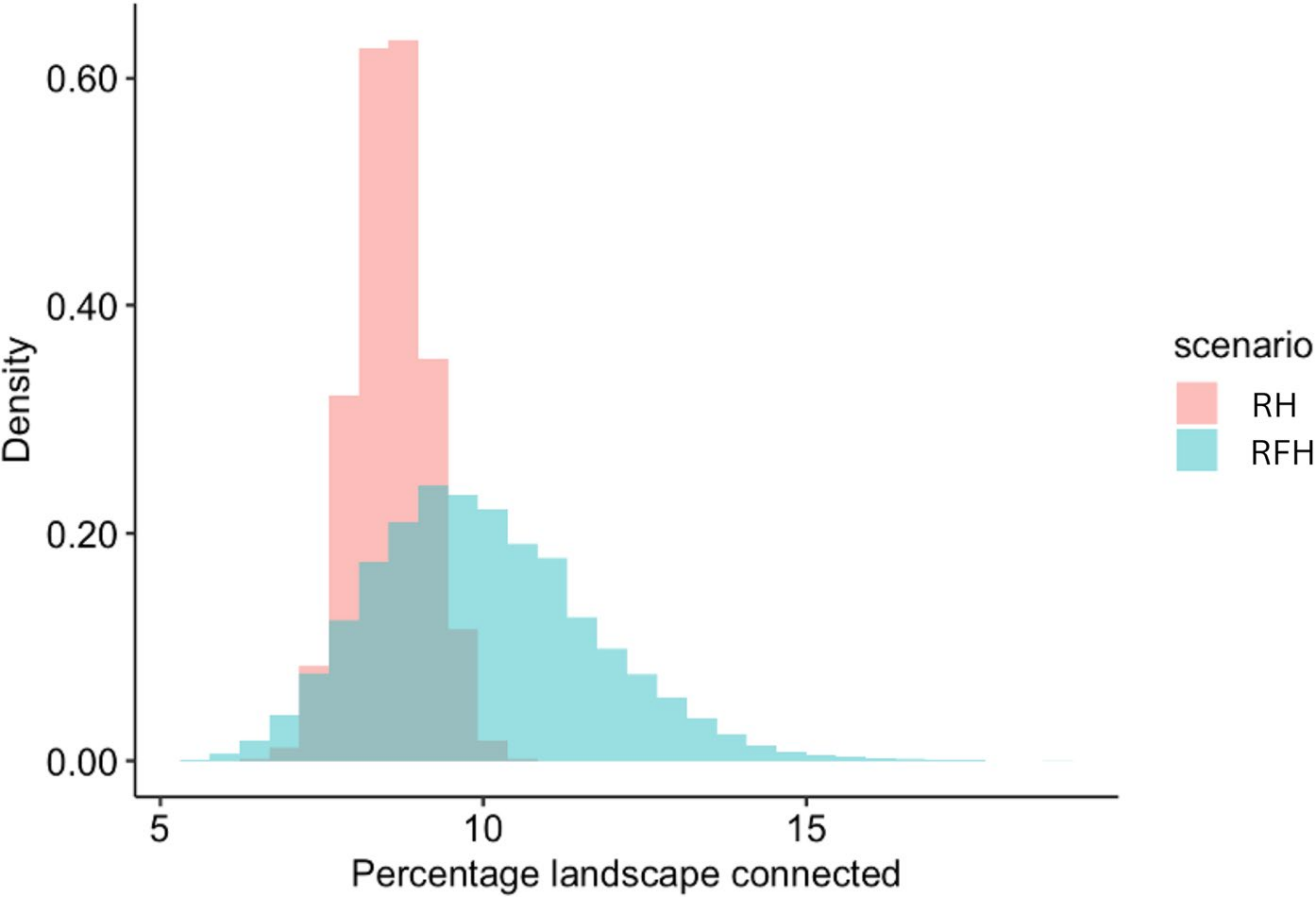
Distribution of reachable habitat (RH: mean = 8.56%, min = 6.28%, max = 10.69%.) and reachable functional habitat (RFH mean = 9.88%, min = 5.33%, max = 18.91%.) values (incorporating negative edge effects)

A complete picture of functionally connected habitat is then possible by creating a distribution of functional habitat and cost values. The distribution of least cost paths between patches is shown in Fig. 10. (where paths are based on functional cost and habitat perspectives). The final distribution for reachable functional habitat suggests a mean value of 9.98% connected habitat, with a minimum of 5.53% and a maximum of 18.91% (Fig. 11), exhibiting a broader distribution than the estimate of reachable habitat based only on membership to the woodland class (“reachable habitat”: RH in Fig. 11)  (mean = 8.56%, min = 6.28%, max = 10.69%). Potential functional connectivity computed for the Boolean landscape in Fig. 6A (computed as EHI without neighbourhood effects and using contiguous patch area as a surrogate for habitat provision) was considerably higher (13%) than the mean estimates for either of the fuzzy approaches. This demonstrates the significance of including neighbourhood effects for a more comprehensive understanding of the distribution of possible paths between habitat patches.

## Discussion

In this first of two papers addressing the challenge of habitat delineation, we have presented a framework that leverages a perspective based on a distribution of potential outcomes as opposed to Boolean conceptualisations of landscape characteristics (see Dennis et al. this issue for a case study application). In so doing, our method provides a flexible approach that addresses a range of issues related to the use of the habitat patch concept and its spatial delineation. We demonstrate how this method can be directly implemented into the analysis of landscape pattern and process relevant to questions around fragmentation-biodiversity outcomes. The habitat patch remains a central concept informing theory and practice within landscape ecology (Kupfer 2012), but the development of metrics that reflect ecological processes continues to be hindered by the limitations of working with contiguous (patch-based) or continuous (gradient-based) approaches (Frazier and Kedron 2017). This issue has been formally described as an instance of the *continuity-contiguity problem* in landscape ecology (Dennis and Huck 2025), the persistence of which prevents also a satisfactory solution to the gap-crossing problem and the problem of multivariate habitat delineation. The methodological approache described here address these problems via three key developments: 1) rejecting the assumption that "habitat area” is always commensurate with "habitat amount”; 2) operationalizing habitat distributions using type-2 fuzzy set theory and 3) employing spatial kernels to model patch-landscape interactions. Spatial kernels have already been used to delineate edge effects and edge-weighted habitat provision (Dennis et al. 2024b) and type*-1I* uncertainty has been explored in the context of land cover classification (Fisher 2010). However, the great potential for leveraging these approaches into a common framework for habitat delineation aimed at modelling ecological processes has, until now, not been realized. This is likely due to the absence of an ecological basis for their implementation, which we provide here. Through the harnessing and integration of these approaches it is possible to go beyond the need for fixed habitat patch boundaries and to achieve a more flexible delineation of habitat. The methodological roadmap provided here contributes to an overall framework for a functional spatial ecology combining geographical information science and landscape ecology principles and reconciling the continuity-contiguity impasse.

### Re-assessing habitat delineation, quantification and fragmentation

Adopting a framework based on a flexible view of functional habitat has significant implications for fields of inquiry concerned with the role of habitat provision and fragmentation on biodiversity outcomes at the landscape scale. This is because the functional habitat perspective takes a view of fragmentation that is derived from functional rather than structural properties of the landscape and the species under investigation. We argue that, given the acknowledged need for functional approaches to landscape processes such as habitat connectivity (Watts and Handley 2010; Keeley et al. 2021), the absence of a functional derivation of habitat itself is conspicuous with implications for how the relationship between fragmentation, connectivity and biodiversity is measured and assessed. For example, from a meta-population perspective, the gap-crossing problem is effectively addressed by the use of dispersal kernels parameterized with species-specific dispersal values in connectivity assessments. However, at the patch-level, characteristic foraging behaviour and the occurrence of resources in the neighbouring landscape may also influence habitat availability but are ignored in existing habitat delineation methods. By including multivariate estimates of habitat and operationalizing neighbourhood effects, the functional habitat perspective permits a more comprehensive view of factors that influence habitat provision at the patch-level. Additionally, by adopting a fuzzy approach to assessing contiguity (patch delineation), our framework provides a more complete understanding of the degree to which the gap-crossing issue affects habitat availability (i.e. at the landscape-level as reachable functional habitat). Hence, these advances explicitly foreground the role of spatial heterogeneity in habitat delineation, a key factor that, whilst central to landscape ecology theory, is largely absent from studies exploring the fragmentation-biodiversity relationship.

Though we focus here on the leveraging of classification uncertainty to delineate habitat patches in a more functional way, the underlying theoretical principles and methodological framework can equally be applied to a range of other applications. For example, *Type 2* fuzzy objects may be derived for single biotopes if required. Similarly, landscape mosaics and gradients could, if desired, be effectively delineated by applying an appropriate alpha-cut (in the case of the former) and the type-1 output (in the case of the latter) for the biotopes thought to comprise these mosaics or characterize gradients of interest. However, such outputs ultimately take on a Boolean description of landscapes and are not the goal of our approach (which rests on a functional view of habitat). For example, if we adopt the functional view, then the existence, or not, of landscape mosaics or of intergrading land covers is only relevant insofar as they represent habitat resources for a given species. In other words, a functionally relevant view of *habitat* mosaics cannot be contained within a single Boolean framing. Rather, we must see that such mosaics emerge as a function of species-environment relationships. Similarly, hotspots of diversity such as ecotones are known to have a disproportionate influence on species composition at the community level but are poorly represented in Boolean classification schemes. If we take a functional view of habitat delineation, however, then this is the area within which suitable (i.e. species-specific) habitat resources consistently arise as per our method. Hence, the contribution of the ecotone is simply the degree to which functional habitat arises for a number of different species within its spatial extent. Note that, from a functional perspective, it therefore makes little sense to consider such habitat as “edge” as opposed to “core” habitat but is simply the spatial extent in which suitable habitat conditions arise. We position this work, therefore, within the wider, critical need for consistency on the use of functional versus structural definitions in landscape ecology. Notwithstanding the theoretical appeal of, and methodological need for, a functional delineation of habitat to better understand species-specific responses to patch dynamics, a Boolean approach to mapping land cover retains its relevance in many practical contexts. For example, field surveys, practical conservation planning and natural resource management inventories will continue to benefit from accurate land cover maps based on Boolean schemes. The unique contribution of our approach rests on the degree to which landscape and restoration ecologists seek to implement a functional view of species-environment responses within research and modelling protocols. Moreover, we do not see such workflows as being mutually exclusive given that both the Boolean and functional approaches can be achieved from the same input model (rather, it is the emphasis on multivariate habitat and model uncertainty that differentiates them).

A potential limitation of any approach to delimiting discrete spatial objects is the sensitivity of measurement to the resolution at which information is sampled, such as the cell size associated with imagery data. However, the fuzzy approach adopted here retains more information than either a Boolean or *type-1* fuzzy classification scheme regardless of the data resolution. Hence, though the issue of measurement precision as a function of data resolution persists, it is mitigated to the extent that our approach leverages the uncertainty in landscape classifications (which is generated in part by sub-pixel heterogeneity) towards a more comprehensive analysis. Hence, what detracts from model performance in conventional classification approaches (where single outcome model results are undermined by uncertainty) becomes a strength when that uncertainty is leveraged to produce a more realistic and comprehensive range of possible outcomes.

### Implications for fragmentation-biodiversity research

At the core of current attempts to understand the role of fragmentation on biodiversity outcomes is the general assumption that habitat area and amount are commensurate (e.g. Evju and Sverdrup-Thygeson 2016; Lindgren and Cousins 2017; Melo et al. 2017; Watling et al. 2020; Zhang et al. 2024). This implies both a homogeneous distribution of resources within and between habitat patches and the absence of any influence of heterogeneity in the neighbouring environment. Hence, current understanding of fragmentation effects may be undermined by a limited view of habitat availability. The framework presented here advances this position by integrating contiguous, continuous and functional properties of habitat that can be used with any of the commonly used fragmentation-related metrics (e.g. patch density, size or isolation). As such, it provides a basis for improved hypothesis testing. For example, the relative importance of fragmentation per se on habitat connectivity is a central, as yet unanswered, question within fragmentation-biodiversity research (Fahrig 2013, 2021; Saura 2021; Watts and Hughes 2024; Riva et al. 2025) with “connectivity” typically described using coarse proxies such as mean Euclidean distance (Fahrig 2021; Riva et al. 2025). However, even if connectivity were to be measured by more functional methods, there is currently no corresponding method to measure fragmentation. This reveals an inconsistency whereby both structural and mechanistic perspectives are employed within fragmentation-biodiversity research. We therefore recommend that the Boolean approach to estimating fragmentation per se be abandoned on the basis that this captures only a structural understanding of fragmentation. For example, effectively operationalising both habitat area and an “appropriate ecological distance” is central to inquiries based on the habitat amount hypothesis (Fahrig 2013; Watling et al. 2020). However, if these are assessed through purely structural (geometric) rather than functional means, results may be misleading without contextual understanding of a species-oriented view of habitat quality and species sensitivity (e.g. to neighbourhood effects). See Dennis et al. (this issue) for further discussion on this point and a demonstration of how structural and functional perspectives shape the observed relationship between fragmentation and habitat connectivity in a real-world example.

## Conclusion

In this first of a pair of papers presenting a new approach to habitat delineation, we have presented an original methodological framework and demonstrated its use in the computation of fragmentation-related metrics leveraging a functional perspective. The key components of this perspective and the associated framework are the (1) the multivariate delineation of habitat based on *type-1* fuzzy membership and the operationalising of neighbourhood effects and (2) the harnessing of uncertainty in land cover classification (*type-2* fuzzy membership) to achieve a distribution of possible outcomes that resolves the *continuity-contiguity problem*. This methodological roadmap provides the foundation of an overall framework for a functional spatial ecology combining geographical information science and landscape ecology principles and reconciling the *continuity-contiguity problem*. The adoption of this approach, emphasising a functional view of habitat and its fragmentation, will have a considerable impact on landscape ecology research by providing a theoretical and methodological means to apply a context-aware perspective to the fragmentation-biodiversity debate (Valente et al. 2023). In the second of this pair of papers (Dennis et al. this issue), we provide an example of how this enquiry may be developed.

## Data Availability

Data and code to implement functional fuzzy habitat delineation presented here are available at the corresponding author’s Github page: https://github.com/dennisMatt/schrodinger
